# M1 a‐tDCS does not acutely enhance motor skill acquisition of a dexterous, timing‐based videogame task in adults

**DOI:** 10.14814/phy2.70978

**Published:** 2026-06-16

**Authors:** Brandon O. Blake, Walter P. Burton, Emelia E. Duchow, Quinn McCallion, Brach Poston, Zachary A. Riley

**Affiliations:** ^1^ School of Health and Human Sciences Indiana University–Indianapolis Indianapolis Indiana USA; ^2^ Department of Kinesiology and Nutrition Sciences University of Nevada‐Las Vegas Las Vegas Nevada USA

**Keywords:** learning, M1, motor skill, tDCS

## Abstract

Motor skill acquisition involves plastic changes across distributed neural networks, and anodal transcranial direct current stimulation (a‐tDCS) applied over primary motor cortex (M1) is often used to modulate excitability and potentially enhance learning. This study examined whether pairing a‐tDCS with practice of a dexterous, timing‐based videogame task would improve performance and retention relative to SHAM stimulation. Forty healthy adults practiced a Guitar Hero–style rhythm game requiring rapid finger sequencing, fine motor control, and temporal precision while receiving either 1 mA a‐tDCS or SHAM stimulation for 20 min. Performance was assessed through accuracy, best continuous note streak, and overstrums across pre‐test, practice, post‐test, and 24‐h retention sessions. Both groups demonstrated significant improvements across all performance measures with practice, and gains were maintained or further enhanced after 24 h. However, no differences were found between the a‐tDCS and SHAM groups at any time point, and no time × group interactions emerged. Overall, a‐tDCS did not enhance skill acquisition or retention, highlighting task specificity as a key factor in neuromodulation efficacy.

## 1 INTRODUCTION

Motor skill acquisition, or the progressive improvement of movement accuracy, speed, and control through practice, is supported by plastic changes across distributed cortical networks (Dayan & Cohen, 2011; Tanaka et al., 2011). Motor skills practiced for proficiency can range from the simple, such as single button pressing, to complex skills requiring multi‐joint coordination, sequencing, and timing. The demands of the motor skill being performed determine the relative contribution of different brain regions involved in supporting improved performance: including, but not limited to, the motor cortex (M1), cerebellum, basal ganglia, and the prefrontal cortex (Huang et al., 2011; Krakauer et al., 2019; Krakauer & Mazzoni, 2011). In simplified terms, tasks that are not heavily reliant on error feedback (e.g. via a perturbation) or demand changing task strategies, will require use‐dependent plasticity from M1 (Krakauer & Mazzoni, 2011). The use‐dependent plasticity in the motor cortex is subject to different types of modulation across fast (online) and slow (offline) stages of learning (Butefisch et al., 2000; Dayan & Cohen, 2011).

The evidence for online learning in M1 comes largely from studies using paired associative stimulation, or stimulation of M1 alone, to enhance M1 activity in a manner consistent with motor task execution (Sanes & Donoghue, 2000; Ziemann, 2004a, 2004b). Specifically, when learning a new motor skill, the repeated activation of task‐specific cortical neurons causes changes in synaptic strength and connectivity, which are expressed as short‐term changes in cortical excitability as measured with transcranial magnetic stimulation (TMS) (Classen et al., 1998; Meek et al., 2021; Tyc & Boyadjian, 2011). Increased cortical excitability in M1 can be driven by increased excitability in one specific area, or an increase in the spread of excitability through transsynaptic connections within a somatotopic region, particularly for the hand muscles (Kleim et al., 1998; Kleim et al., 2002; Nudo et al., 1996). Though this effect is considered transient (Monfils et al., 2005), it may play an important role in the degree of retention of the motor skill, which we would subsequently generalize as learning (Cantarero et al., 2013; Spampinato & Celnik, 2017).

M1 has a complicated role in learning of motor skills because it may not be the sole region of the brain responsible for immediate performance improvements during an initial practice session. The cerebellum likely drives these changes due to the role it plays in assessing movement error that is used to correct trial‐by‐trial learning (Penhune & Steele, 2012; Smith & Shadmehr, 2005). However, sufficient activation of M1 during the initial trials or practice is critical for triggering processes that lead to task and performance retention. Many studies have demonstrated that occluding long‐term potentiation (LTP) and increasing long‐term depression (LTD) in M1 is what permits motor learning to occur (Rioult‐Pedotti et al., 2000; Rioult‐Pedotti et al., 2007; Rosenkranz et al., 2007). Strengthening the appropriate synapses needed for performing a specific task through LTP/LTD increases the capacity for modulating M1 plasticity, and subsequently the amount of retention from each session (Cantarero et al., 2013). Thus, increases in M1 excitability, session after session, should result in more retention and greater task learning and performance over time.

Anodal transcranial direct current stimulation (a‐tDCS) has emerged as a portable, well‐tolerated neuromodulation method to change cortical excitability via subthreshold polarization of neuronal membranes (Buch et al., 2017; Paulus, 2011). Several studies have demonstrated that stimulation duration/intensity can scale the magnitude and persistence of excitability changes, with downstream impacts on behavior (Nitsche & Paulus, 2000; Stagg et al., 2018). Mechanistically, a‐tDCS can modulate resting membrane potential, synaptic efficacy, and network connectivity, producing acute and longer‐lasting changes that share features with LTP/LTD‐like plasticity (Antal et al., 2011; Bikson et al., 2016; Qi et al., 2024; Yamada & Sumiyoshi, 2021). Specifically, M1 has been a target of a‐tDCS in many studies and has clearly been shown to increase cortical excitability in this region with as little as 10 min of stimulation and across a range of stimulation intensities (Kidgell et al., 2013). When a‐tDCS is combined with physical training, cumulative findings indicate greater motor‐evoked potentials (MEPs), faster reaction times, and reduced errors than training alone, supporting a synergistic neuromodulation‐plus‐practice approach (Ammann et al., 2016; Ehsani et al., 2025; Halakoo et al., 2020). Taken together with the preceding evidence, increased cortical excitability from practice of a task, paired with a‐tDCS applied to the region, should result in greater learning and retention than just from practice alone.

Previous work from our laboratory has shown mixed results with the application of a‐tDCS to M1 when practicing different motor tasks. For instance, M1 a‐tDCS did not result in accelerated learning of a simple choice reaction time task (CRT) (Wilson et al., 2025), or when throwing darts to randomly selected targets (Perez et al., 2025). However, we have observed improved performance with M1 a‐tDCS, relative to SHAM (no stimulation), with a tweezer dexterity task (Wilson et al., 2022), and with a videogame task that required relatively complex rhythm and timing of key pressing (Meek et al., 2024). Both tasks required significant dexterity with the digits and some degree of motor sequencing, though the Meek et al. study also depended on temporal precision.

The original videogame “Guitar Hero” provides a compelling model for studying skill acquisition and retention, leading to its use in motor training studies (Ngomo et al., 2012). Video games are a useful tool for understanding and quantifying the learning of dexterous tasks (Greenwell et al., 2025; McCallion et al., 2025; Meek et al., 2024; Stafford & Vaci, 2022). Using a guitar‐like controller to interact with the videogame makes the game a dexterous sequencing task that necessitates rapid action selection, fine motor control, timing precision, and can afford direct measurements of performance and learning.

The purpose of the present study was to evaluate whether a‐tDCS applied over M1 during practice of a dexterous videogame task produces larger improvements in performance than SHAM (no stimulation) and whether these improvements persist offline and can be retained 24 h later. By quantifying skill via videogame performance, the study aims to clarify whether a‐tDCS of M1 primarily modulates fast learning and/or the initiation of slow learning. A final aim is to contribute a repeatable tDCS protocol that can add to the pre‐existing literature base on this widely used, yet not clearly understood, neuromodulation technique.

## 2 MATERIALS AND METHODS

### 2.1 Participants

Forty healthy subjects (22 male; age 22.0 ± 3.1 years) participated in the study. The Indiana University Human Subjects IRB Committee approved the study (#26243) and all subjects provided written consent before participating. Study procedures were in accord with the Declaration of Helsinki. Subjects completed a checklist to self‐report that they had no neurological disorders, recent history of injury or disease involving the upper limbs, history of seizures or epilepsy, psychiatric disease, and no pacemaker or other metal implants in the upper body. Subjects also completed an Edinburgh Handedness Inventory (Oldfield, 1971). Only 2 of the 40 subjects were identified as being left‐handed by the handedness inventory. Participants were asked to abstain from caffeine or stimulants (i.e. coffee, pre‐workout supplements, soda, energy drinks, tea, nicotine or tobacco products, etc.) for at least 12 h before their visits. Additionally, subjects who were prescribed and currently using medications for the treatment of attention deficit disorder (ADD) and/or attention deficit hyperactivity disorder (ADHD) were excluded from participation in this study. All subjects completed a brief gaming history questionnaire, and subjects were excluded if they had a history of playing a stringed instrument (e.g. guitar) or videogames using a guitar controller.

### 2.2 Experimental timeline

**FIGURE 1:**
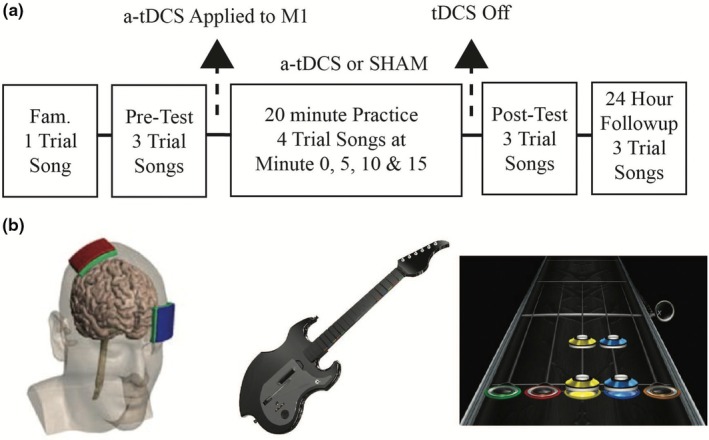
(a) Depicts the timeline of procedures for the testing sessions, including the number of trial songs and the application of a‐tDCS. (b) Head model showing the electrode montage for tDCS over M1 and the supraorbital region. A picture of the guitar‐like controller used to interact with the videogame in the study. Also shown is a picture of the gaming screen, as viewed by the subjects during gameplay.

The subjects were randomly assigned to either SHAM (control; *n* = 20) or anodal‐tDCS (a‐tDCS; *n* = 20) stimulation groups. Participants were randomly assigned to two groups using a computer‐generated random number sequence, and the order of conditions was counterbalanced across groups to control for order effects. This was a single‐blinded design, so only the participant was blinded to the group they were assigned to. They visited the lab on two occasions at approximately the same time of day (±2 h). The experimental task involved subjects playing a scrolling videogame with a guitar‐shaped controller. In total, each subject completed a familiarization trial, and then pre‐test, practice, post‐test, and one retention block (24 h follow‐up) of the videogame task, with the only difference being the stimulation condition (Figure 1a).

### 2.3 Videogame task

The videogame used for the study was Clone Hero v1.0.0.4080, which is an open‐source instrument‐based rhythm game, similar to the mass‐produced videogame Guitar Hero. The controller used in the study was an 8.2 × 54.3 × 26.7 cm PDP Riffmaster Wireless Guitar Controller (Turtle Beach, San Diego, CA, US). The subjects' left hand, also referred to as their fret hand, held the neck of the controller with their left thumb wrapped around the back of the neck for support, while their other four digits rested on the first four fret buttons. The right hand (strumming hand) was stationed just in front of the controller, with the right thumb gently rested on the strum bar. Briefly, the object of the game is to strum the guitar controller while simultaneously holding down the correct button on the fretboard. Different colored notes (green, red, yellow, and blue) corresponded to specific digits on the participants' left hand (II–V). For example, during gameplay when the yellow and blue notes overlapped with the yellow and blue strumming queues, participants would need to simultaneously press the yellow and blue fret notes on the controller with their ring and pinky fingers (digits IV and V) while also pushing down on the strum bar with their right thumb. As different combinations of notes scroll upward on the virtual fretboard screen, this process was reproduced for the different colors and corresponding digits (Figure 1b). When the subject strums while holding the correct button down, when the notes overlap on the screen, the input is registered as a successful note played. For the purposes of this study, only the first four buttons, or “frets” were used, corresponding to use of Digits II–V (index—pinky fingers). All the subjects used a traditional guitar playing posture; seated, with the controller in their lap, the dominant hand strumming and the non‐dominant hand pushing buttons on the fretboard.

Each subject did a single song trial as a familiarization. After that, they completed 3 of the same song trials as the pre‐test block. A rest duration of 30s was provided between song trials. The practice block was 20 min in duration and required the subjects to perform 4 song trials at minutes 0, 5, 10, and 15 of the practice time block. The same 3 song trial block was performed as the post‐test, immediately after the 20 min practice period. Subjects then came back to the lab after 24 h to repeat the 3 song trial block as a measure of skill retention.

All subjects played the familiarization trial on the easy setting. In this setting, the trial song had 347 notes to play over ~3.5 min. If the subject scored greater than 80% of the notes hit in the familiarization trial, the difficulty setting was increased (440 notes over the same duration) and they repeated the trial. If they scored above 80% again, it was increased one more time (562 notes over the same duration). Only 7/40 subjects played the remainder of the trials at 440 notes, and 1 subject played at 562 notes. This scaling of difficulty was done to avoid ceiling effects in learning the videogame, as we would expect a compressed range of improvement if they started with >80% accuracy. The subjects that required additional familiarization trials were provided more overall practice, though the increased speed of the successive trials should have limited the benefit of added repetitions.

### 2.4 Brain stimulation

Participants were randomized to M1 a‐tDCS and SHAM stimulation groups. This was a single‐blind study design as only the subject was unaware of the stimulation condition. Both groups underwent the same preparation of the a‐tDCS, however, the SHAM condition simulated the sensation of stimulation by incorporating a brief ramp up (30s) to 1 mA followed by the cessation of stimulation at the beginning of the practice period and a brief ramp down (30s) at the end of the 20‐min session, with no current being delivered outside of the ramp times. None of the subjects had experienced a‐tDCS prior to participating. For both groups, saline soaked sponges were placed on the scalp and stimulation was delivered with a Soterix 1 × 1 tDCS Device (Soterix Medical, NY). A 35cm^2^ target electrode was fixed over the motor‐cortical position for the non‐dominant arm C3 or C4, International 10–20 System; (Klem et al., 1999), and another 35cm^2^ return electrode was placed on the ipsalateral supra‐orbital area (Fp1 and Fp2 according 10–20 EEG Coordinate System). All electrodes were secured with straps to prevent movement during the task. The current was delivered through the electrodes at a constant current of 1 mA for 20 min during the entire practice block of the task. The stimulation intensity of 1 mA was chosen since it has been shown to provide the most reliable change in motor cortex excitability and is at the low‐end of the range of intensities that is considered safe with a‐tDCS (Ammann et al., 2016; Antal et al., 2025). Current density magnitude (A/m^2^) was computed using finite‐element modeling in SimNIBS as J = σ·E. Values were averaged within gray matter regions of interest beneath the anodal M1 electrode. Mean gray‐matter current density beneath the anode was 0.028 A/m^2^ (95th percentile: 0.11 A/m^2^) (Thielscher et al., 2015), which is the same as used by other recent studies in our lab (Meek et al., 2024; Perez et al., 2025; Wilson et al., 2025). Finally, no adverse effects to brain stimulation were described by the subjects or noted by the authors.

### 2.5 Data analysis

A priori sample size estimation was performed for a mixed repeated‐measures ANOVA with Group (2 levels) as a between‐subjects factor and Time as a within‐subjects factor. Based on prior data and the limited piloting we performed, we anticipated a mean difference of approximately 25% with a within‐subject standard deviation of 20%, corresponding to a standardized effect size of *d* = 0.75 (Cohen's *f* = 0.38). Assuming *α* = 0.05, power = 0.80, and moderate within‐subject correlations, power analysis indicated that approximately 15–18 participants per group would be sufficient to detect a Group × Time effect. Twenty subjects were ultimately collected per group to ensure sufficient power with unexpected variability.

Three different measurements were included in the analysis of videogame performance. Specifically, the analyzed game metrics included accuracy (which is a measure of notes hit/total notes, total number of notes hit), best continuous streak, and overstrums (which records missed attempts to hit the note). Each of these measures was averaged across three complete song trials for the pre, post, and follow‐up testing. The practice block was different, as the videogame performance measures were averaged across 5 song trials during the 20 min practice period. Mixed model ANOVAs were used to compare accuracy, best streak, and overstrums between the SHAM and a‐tDCS groups with repeated measures across the time points (within‐subjects 4 time points, between‐subjects 2 groups [a‐tDCS & SHAM]). In cases where Mauchly's test indicated a violation of sphericity, Greenhouse–Geisser corrections were applied to adjust the degrees of freedom. All analyses were conducted in SPSS v.29, and a significance level of *p* < 0.05 was used.

## 3 RESULTS

**FIGURE 2:**
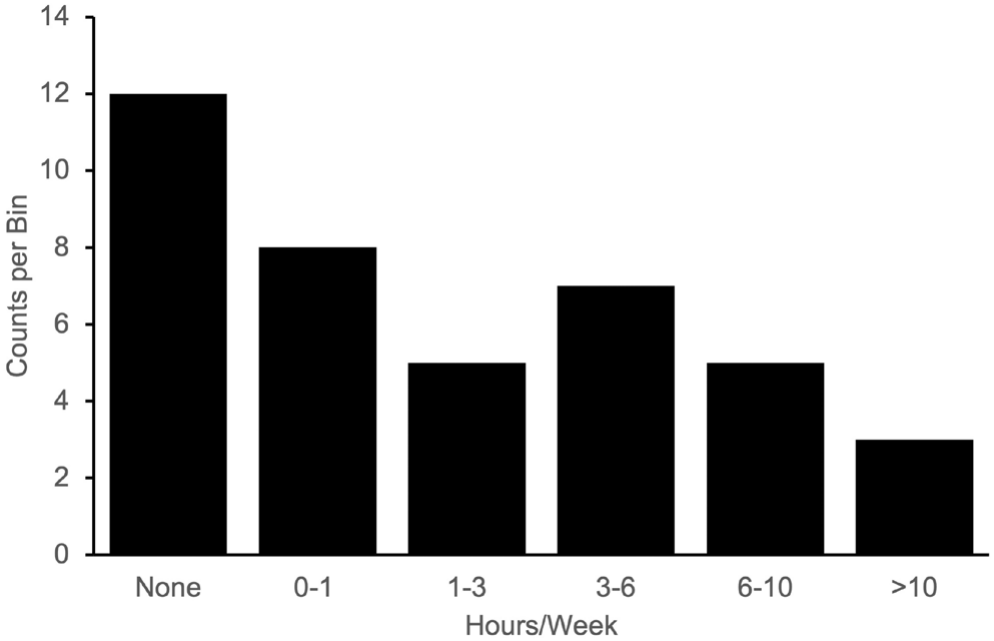
Histogram showing the amount of weekly videogame play for the subjects included in the study. Ranges were provided to subjects and they indicated which bin they best fit.

Of the 40 participants included, their weekly gaming experience was: 0 h (*n* = 12), 0–1 h (*n* = 8), 1–3 h (*n* = 5), 3–6 h (*n* = 7), 6–10 h (*n* = 5), and >10 h per week (*n* = 3, Figure 2). Importantly, there were no differences in weekly gaming activity between the two groups (*t* = −0.907, *p* = 0.376).

**FIGURE 3:**
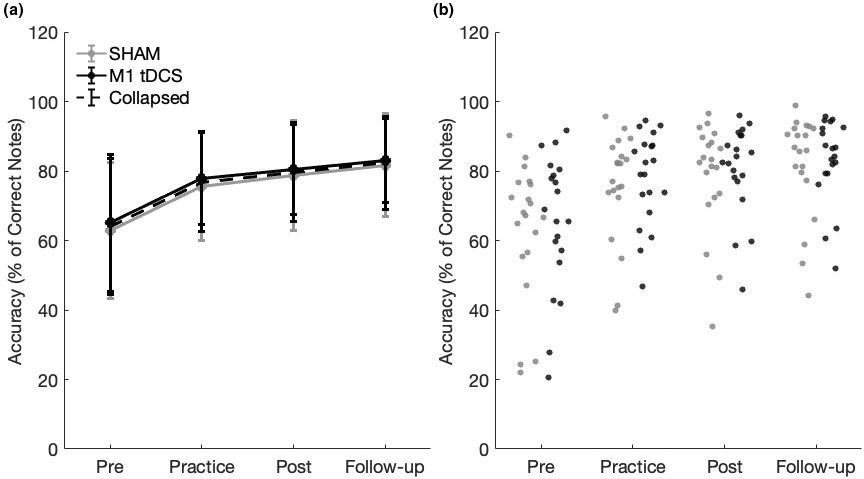
(a) The accuracy of the subjects, expressed as a percentage of the correct notes played, across the four time periods. Lines are included for the a‐tDCS group, SHAM group, and the dotted line represents all subjects collapsed into a single group. Error bars represent standard deviation. (b) Grouped scatterplots showing individual data for SHAM (gray dots) and a‐tDCS (black dots) across the four time periods.

**FIGURE 4:**
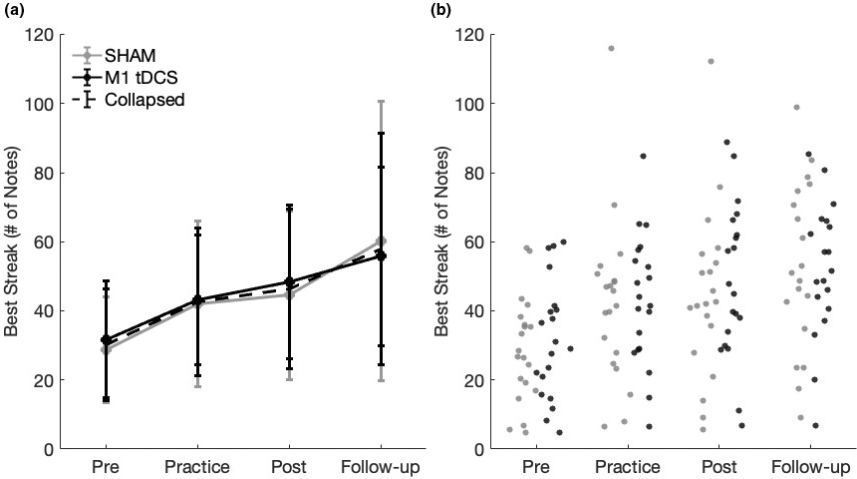
The average best continuous streak for all subjects averaged across the four time periods. Lines are included for the a‐tDCS group, SHAM group, and the dotted line represents all subjects collapsed into a single group. Error bars represent standard deviation. (b) Grouped scatterplots showing individual data for SHAM (gray dots) and a‐tDCS (black dots) across the four time periods.

**FIGURE 5:**
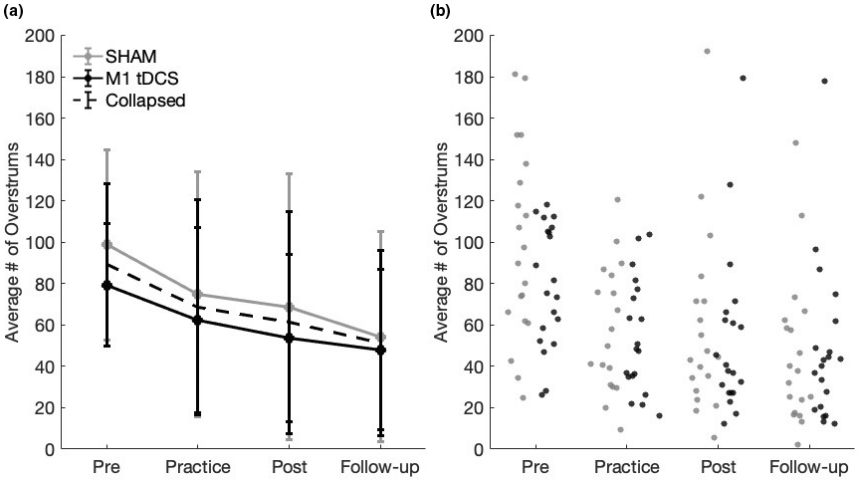
The average number of overstrums for all subjects averaged across the four time periods. Lines are included for the a‐tDCS group, SHAM group, and the dotted line represents all subjects collapsed into a single group. Error bars represent standard deviation. (b) Grouped scatterplots showing individual data for SHAM (gray dots) and a‐tDCS (black dots) across the four time periods.

There was a significant main effect of time across all three videogame measures, showing that both groups increased accuracy (*F* = 74.618, *p* < 0.001, ηp^2^ = 0.801; Figure 3), best continuous streak (*F* = 41.964, *p* < 0.001, ηp^2^ = 0.525; Figure 4), and reduced the number of overstrums (*F* = 31.266, *p* < 0.001, ηp^2^ = 0.451; Figure 5) with practice. However, there were no differences observed between the two groups for any of these performance measures (*p* = 0.391–0.903). Similarly, there were no time × group interactions (*p* = 0.413–0.932). As there were no differences between groups in the variables, Bayesian independent‐samples tests were conducted at each time point using a Jeffreys–Zellner–Siow (JZS) prior (*r* = 0.707). Bayes factors quantified evidence for or against group differences. The Bayes factors for each variable and across each of the four time points were between 0.66 and 0.98, further indicating there were no differences between SHAM and a‐tDCS groups. Following up on this, we also ran TOST (two one‐sided tests) procedures on the percent change in accuracy from the pre‐post tests to determine if the groups were equivalent. The two one‐sided tests procedure did not support equivalence. The 90% confidence interval (−21.07 to 19.47) was substantially wider than the predefined equivalence bounds, indicating a high degree of uncertainty in the estimated effect.

As there were no group differences, we ran individual one‐way ANOVAs on each of the variables collapsed between the two groups to perform post‐hoc tests. For all subjects, accuracy was lower in the pre‐test than the practice (*p* = 0.016), posttest (*p* = 0.001), and follow‐up (*p* < 0.001), and there were no other significant differences between time points. The best continuous streak was significantly lower in the pre‐test (*p* < 0.001) and the practice period (*p* = 0.035) than the follow‐up time. Finally, the number of overstrums was significantly higher in the pre‐test (*p* = 0.009) than in the follow‐up time.

## 4 DISCUSSION

The main purpose of the present study was to determine whether a‐tDCS applied over M1 during practice of a dexterous videogame task resulted in larger improvements in performance than SHAM (no stimulation), and to that aim, no differences were observed between the two groups. Subjects with a diversity of gaming experience were examined, though the two groups were not different in their weekly gaming activity. Furthermore, all subjects improved at the novel videogame task, as evidenced by their improvement in accuracy, best continuous streak of notes hit, and limiting the number of erroneous overstrums. The collapsed data showed that all subjects improved from the pre‐test, and sustained or even continued to improve slightly in a follow‐up session 24 h after practice.

The present study examined whether pairing anodal transcranial direct current stimulation (a‐tDCS) over primary motor cortex (M1) with practice of a dexterous, timing‐based videogame task would enhance motor skill acquisition and retention relative to SHAM stimulation. Our hypothesis was formed from extensive evidence that motor skill learning relies on plastic changes distributed across cortical and subcortical networks (Dayan & Cohen, 2011; Krakauer et al., 2019), and that tasks emphasizing fine motor control and sequencing should draw heavily on use‐dependent plasticity mechanisms within M1 (Krakauer & Mazzoni, 2011). Additionally, a‐tDCS has been widely documented to transiently increase cortical excitability (Meek et al., 2024; Nitsche & Paulus, 2000; Sato et al., 2024; Stagg et al., 2018), thereby theoretically amplifying the M1 circuits used during skill practice. However, we observed no differences between a‐tDCS and SHAM stimulation on any performance metric—accuracy, best continuous streak, or overstrums—either during practice or across early (post‐test) and delayed (24 h) retention. Instead, all subjects showed improvements with practice alone, consistent with typical learning for novel motor skill acquisition.

These findings highlight several important considerations for interpreting the role of M1 in learning tasks that require both dexterity and temporal precision. Although M1 contributes to the strengthening and refinement of task‐specific movement representations, its involvement is nuanced across stages of learning (Butefisch et al., 2000; Spampinato & Celnik, 2017). Early performance changes, such as rapid reductions in errors or improved timing, are often attributed to cerebellar mechanisms governing error‐based updating (Huvermann et al., 2025; Penhune & Steele, 2012; Popa et al., 2016; Smith & Shadmehr, 2005). Consequently, even if a‐tDCS elevates cortical excitability in M1, this may not be the relevant mechanism influencing initial performance gains if there are other parallel changes in the brain. The current videogame paradigm may be a good example of this, where the task requires fine control of finger movements, but the continuous, scrolling rhythm and requirement for moment‐to‐moment timing accuracy may place a substantial burden on cerebellar predictive processing rather than on M1‐mediated use‐dependent plasticity.

Contextualizing the current results within our laboratory's prior work supports the mixed effectiveness of M1 a‐tDCS across tasks with differing motor demands. Tasks requiring prominent fine‐motor precision and sequencing, such as tweezer dexterity (Wilson et al., 2022), or rhythm‐timing gameplay with substantial temporal regularity (Meek et al., 2024), show performance enhancement with a‐tDCS. In contrast, a‐tDCS failed to improve performance on simpler or more decision‐based tasks, such as choice reaction time (Wilson et al., 2025) or variable‐target dart throwing (Perez et al., 2025). The present findings align more closely with these latter results, suggesting that not all dexterous tasks engage the specific M1‐dependent plasticity mechanisms that a‐tDCS is capable of modulating. Although the Guitar Hero videogame task superficially resembles the rhythm‐timing task from Meek et al. (2024), two key differences may reduce the likelihood of M1‐specific enhancement: (1) the Guitar Hero task requires bimanual activation as one hand is “strumming” while the hand subjected to a‐tDCS is pushing down frets, and (2) the Guitar Hero task can require two notes (fret buttons) to be held down simultaneously, whereas the rhythm‐timing task from Meek et al. only required pressing one arrow key at a time. These features may have shifted computational demands of coordination toward cerebellar and fronto‐striatal circuits that are not influenced by a‐tDCS M1 stimulation.

Another potential explanation involves ceiling effects or task difficulty optimization. While difficulty scaling was implemented to avoid excessively high starting accuracy, several subjects still performed above 70%–80% accuracy early in training. Tasks with constrained error margins may leave limited space for stimulation‐related improvements, particularly given that a‐tDCS often produces modest behavioral effects even under ideal conditions (Ammann et al., 2016; Buch et al., 2017). Furthermore, the 20‐min practice block may not have been sufficient to fully engage the slower, consolidation‐related processes where M1 excitability changes can have a stronger influence (Cantarero et al., 2013; Ohashi et al., 2019). The lack of group differences at the 24‐h retention test reinforces this interpretation, as both groups retained their gains but showed no divergence in skill consolidation trajectories. Of note, the only exclusion criteria was that the subjects had not previously played the videogame or real stringed instruments. We did not stratify or exclude anyone based on their use of other fine‐motor skills (e.g. keyboard typing), and this could have contributed to the lack of group differences.

The null findings also raise methodological considerations regarding stimulation intensity, polarity, electrode montage, and timing related to training. Although 1 mA stimulation reliably increases M1 excitability (Kidgell et al., 2013), recent work suggests that montage‐specific current flow patterns, baseline excitability state, and individual neuroanatomical variability all moderate the behavioral effectiveness of tDCS (Antal et al., 2025; Bikson et al., 2016). In a heterogeneous sample of subjects with variable gaming backgrounds, these individual factors may have introduced response variability that masked group‐level differences. The additional testing of equivalence using TOST procedures further demonstrated the high degree of uncertainty in the estimated effect. Regardless of sample size, the variability in individual responses to playing Guitar Hero makes distinguishing group changes (or equivalence) nearly impossible. Based on observations during the study, some of this may be due to how easy it is to get thrown off rhythm or deviate from the required notes for entire, long sequences, even in high‐performing individuals.

Finally, these results contribute to ongoing discussions about reproducibility and task‐specificity in the tDCS literature. Although a‐tDCS can enhance motor‐evoked potentials and facilitate learning in some paradigms, the effects are not universal. Our findings add to recent reports emphasizing that benefits from tDCS are contingent on the stimulation site, neural systems actively engaged by the task, and the type of learning being measured. The present study thus provides an important datapoint demonstrating that, for complex dexterous tasks with substantial timing demands, enhancing M1 excitability alone may not be sufficient to modify performance or learning.

### 4.1 Limitations

Several potential limitations have already been discussed, including the number of subjects, the difficulty with optimizing and standardizing the task difficulty, determining the proper exclusion criteria, and issues related to tDCS (montage, duration, intensity). Statistically, this study is difficult to interpret because the two groups were not close to being different, yet were also far off from being equivalent, due to the high variability from trial to trial. As this study was exploratory in nature, with a novel task, we expected to have some of these issues with variability. It should also be noted that this is a bimanual task. We were exploring the use of tDCS on the hemisphere corresponding to the ‘fret’ hand while not considering the strumming hand or the hemisphere corresponding to that. It is possible that the best use of tDCS is some combination of stimulation sites that utilize both hemispheres. Regardless of the limitations, the task employed in this study provides an interesting model for studying motor skill learning and needs to continue to be explored.

In conclusion, while both a‐tDCS and SHAM groups exhibited clear improvements in videogame performance and retained these improvements after 24 h, a‐tDCS did not confer any behavioral advantage. These results suggest that the specific combination of motor, timing, and sequencing demands in this task may not rely predominantly on M1‐mediated plasticity processes that are sensitive to a‐tDCS. Future work should consider targeting different nodes of the motor learning network, such as the cerebellum or prefrontal regions, or using combined approaches to modulate the neural substrates underlying complex motor skill acquisition.

## AUTHOR CONTRIBUTIONS

**Brandon O. Blake:** Conceptualization; data curation; formal analysis; investigation; supervision; visualization. **Walter P. Burton:** Conceptualization; data curation; formal analysis; investigation; methodology; project administration. **Emelia E. Duchow:** Conceptualization; formal analysis; investigation; methodology; project administration. **Quinn McCallion:** Formal analysis; investigation; methodology; project administration; visualization. **Brach Poston:** Conceptualization; data curation; formal analysis. **Zachary A. Riley:** Conceptualization; data curation; formal analysis; investigation; methodology; project administration; resources; visualization.

## FUNDING INFORMATION

There was no funding provided for this study.

## CONFLICT OF INTEREST STATEMENT

There were no conflicts of interest and no disclosures.

## Supporting information


Data S1.

## Data Availability

Data will be made available upon request. A checklist based on the RATEs checklist (Nejati et al. 2026) is available as Data S1.
