# Supplementary material for: M1 a‐tDCS does not acutely enhance motor skill acquisition of a dexterous, timing‐based videogame task in adults
Source: Physiol Rep. 2026 Jun 16;14(12):e70978. doi: 10.14814/phy2.70978 (PMC13273025; doi:10.14814/phy2.70978)
Supplement: Supplementary file 1 — Data S1. [file PHY2-14-e70978-s001.docx]

Sample Size (Dx), Recruitment Process, Medication: 40 Healthy, No ADHD Med

Age(yrs), Sex(F:M), Edu(yrs), Handedness(R:L:D) : 22 male; age 22.0 ± 3.1 yrs, 38RH

Hours of Sleep, Consumption of Caffeine, Nicotine, and Alcohol: No caffeine/alcohol

Participant Eligibility Criteria: no neurological disorders, recent history of injury or disease involving the upper limbs, history of seizures or epilepsy, psychiatric disease, and no pacemaker or other metal implants in the upper body

Stimulator: Soterix 1x1 tDCS Device

Stimulator:

Sham Option-Yes

Output Channels-1;

Waveform-N/S

Stimulator Safety Features- N/S

Current Resolution-N/S

Monitoring and Feedback-N/S

Electrodes Positioning - C3 or C4, Fp1 or Fp2 International 10-20 System

Inter-electrode Distance- N/S

Shape-Rectangle

Assembly -N/S

Size – 7x5cm 35^2

Contact Medium- Saline

Orientation -N/S

Impedance – N/S

Material-N/S

Connector Position -N/S

Number: one pair

Montage:

Current Intensity (mA) -1

Amplitude Density (V/m) -0.028 A/m² (95th percentile: 0.11 A/m²)

Personalization- N/S

Distribution (Method) -N/S

Duration (min) -20

Frequency (Hz) -N/S

Ramp up/down (sec) -30s

Polarity -N/A

Warm-up time (min) -None

Waveform -N/S

Sham Characteristics- 30/30

Procedure Study Setting and Site

Attrition (n) =0

Hypothesis Statement- Confirmatory

Blinding Method- single

Preregistration – N/S

Ethical Considerations- Yes

Session Duration (min) – 1:30, 20 min tDCS

Safety Monitoring – Yes

Total Number of Sessions -2

Informed Consent Process- Yes

Session Frequency -24 hours

Conflict of Interest -no

Concurrent Intervention -N/A

tES operator -N/S

Randomization (Method) -Random Number Generator

Data Analysis Plan -Mixed Methods ANOVA

Counterbalancing -Yes

Data Availability -Yes

Study Design: randomized, single blinded

Stimulation and Assessment Task Timing: N/A

Inter-session Interval: 24 hours

Data Collection Time Points: Through, Post

Baseline Assessment: gameplay outcomes

Control Intervention: Sham stimulation

Outcome Measure: Gameplay outcomes
